# The popliteomeniscal fascicles: from diagnosis to surgical repair: a systematic review of current literature

**DOI:** 10.1186/s13018-021-02290-z

**Published:** 2021-02-20

**Authors:** Alessio D’Addona, Andrea Izzo, Giovanni Di Vico, Donato Rosa, Nicola Maffulli

**Affiliations:** 1 A.O.U. Federico II, Department of Public Health, Section of Orthopaedics and Trauma Surgery, Via S. Pansini 5, 80131 Naples, Italy; 2 Humanitas Clinical and Research Center-IRCCS, Via Alessandro Manzoni 56, 20089 Rozzano, MI Italy; 3 Department of Orthopaedics and Trauma Surgery, Clinica San Michele, Maddaloni, CE Italy; 4 Department of Musculoskeletal Disorders, University of Salerno, Salerno, Italy; 5 Centre for Sports and Exercise Medicine, Mile End Hospital, Barts and The London School of Medicine and Dentistry, 275 Bancroft Road, London, E1 4DG UK

**Keywords:** Popliteomeniscal, Fascicles, Postero-lateral corner, Lateral meniscus, Arthroscopy, Repair

## Abstract

**Background:**

Popliteomeniscal fascicles (PMF) are considered the posterolateral meniscocapsular extensions which connect the lateral meniscus to the edge of the tibia. PMFs disruption leads to hypermobility of the lateral meniscus with pain and locking sensation. Recognition and treatment of PMFs tear remain very challenging. The aim of this systematic review is to collect and analyse the articles concerning popliteomeniscal fascicle disruption from diagnosis to surgical approach.

**Methods:**

PubMed, Scopus, Web of Science and EMBASE were searched. Various combinations of the keywords “Popliteomeniscal Fascicles”, “Lateral Meniscus”, “Popliteal Hiatus”, “Posterolateral Corner”, “Tear” and “Surgical Repair” were used. The original literature search identified a total of 85 articles comprising of duplicates. The PRISMA guidilines were followed. Studies in English language and published in peer-reviewed journals were included. Articles with level of evidence I to IV were included

**Results:**

A total of three articles were included in the qualitative analysis. All the articles included are retrospective case series, with a level of evidence IV. Studies concerning patients with pre-operative imaging MRI and clinical assessment, reporting surgical technique and clinical outcomes assessed by physical examination and/or subjective evaluation scales were analysed.

**Conclusions:**

MRI and the Figure-4 test allow to assess PMF tears pre-operatively. Arthroscopic evaluation constitutes the gold standard to confirm the diagnosis. Although surgery is considered resolutive for symptoms, there is still controversy about the most appropriate technique. Further higher quality studies are required.

## Background

**Fig. 1:**
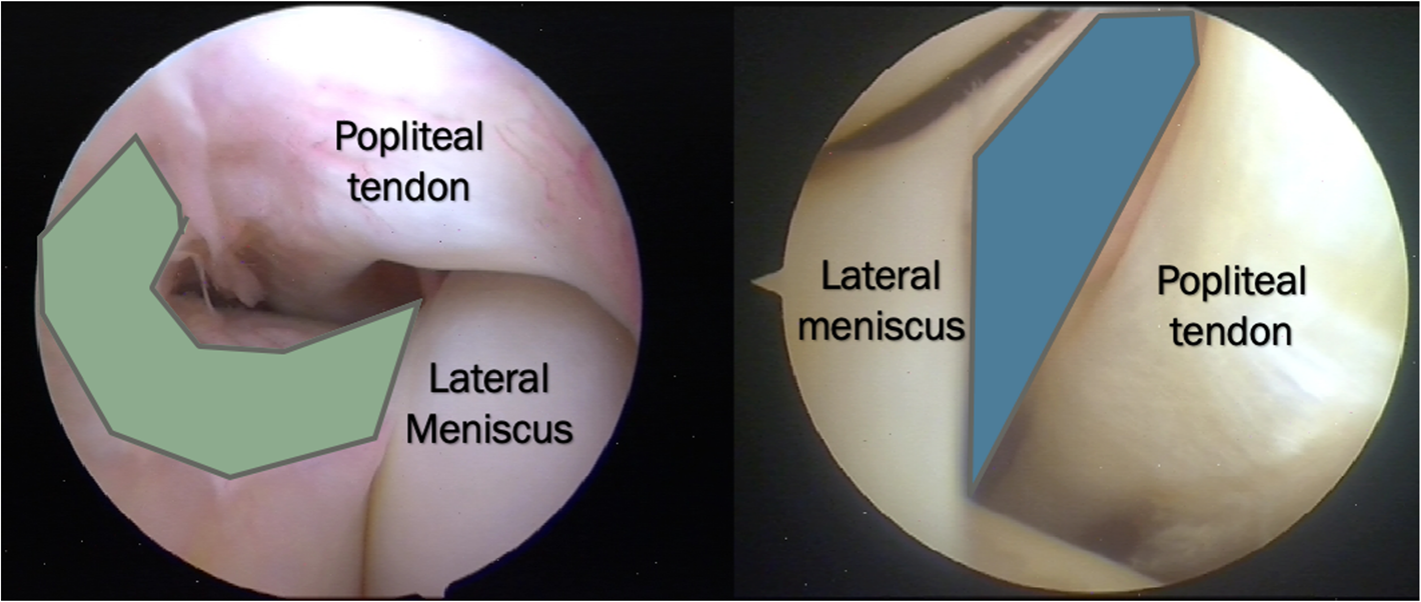
**a**, **b** Arthoscopic anatomy of the PMFs. On the left, in green, it is underlined the antero-inferior fascicle (aPMF). On the right, in blue, the postero-superior fascicle (sPMF) is underlined. It is clear the connection between the posterior horn of lateral meniscus and the popliteal hiatus mediated by the PMFs

The anatomy of posterolateral corner (PLC) of the knee is complex and, given the variable injury patterns, controversy and confusion abound [1, 2]. The PLC is composed of several structures, including the lateral meniscal wall, the popliteus muscle with its tendon and the arcuate popliteal ligament. All of them are reinforced by the deep lateral collateral ligament [3]. The popliteomeniscal fascicles (PMFs) are one of the several structures of the PLC [1, 4, 5]. They are considered the posterolateral meniscocapsular extensions directed inferiorly that allow the popliteal tendon to pass from an intra-articular to an extra-articular compartment [4, 6]. PMFs are composed of two distinct fascicles, the antero-inferior (aPMF) and the postero-superior fascicle (sPMF). The superior fascicle arises from the medial fibers of the aponeurosis of the popliteus tendon, and the inferior fascicle is a coronary ligament which connects the meniscus to the edge of the tibia [7]. A third incostant postero-inferior fascicle is sometime present [8–12]. PMFs, connecting the lateral meniscus at the popliteal hiatus [13], are thought to provide stability to the lateral meniscus, stabilizing the joint during internal rotation of the tibia and sudden changes of direction (Fig. 1) [4, 14–17]. In particular, working in conjunction with the popliteus musculotendinous unit, the PMFs prevent excessive lateral meniscal movement and possible entrapment [8]. For this reason, athletes, such as martial artists, dancers, wrestlers and football players, whose activity is characterised by sudden changes of direction, rotational stresses, repetitive twisting and high jumps, are at higher risk of injuring the PMFs [3, 8, 18]. The injuries which affect the lateral meniscus lead to an increase of contact pressure and rotational instability, predisposing the joint to osteoarthritis as observed on radiographs [1, 19]. A hypermobile lateral meniscus (HLM) may cause knee pain and a locking sensation during deep knee flexion. One of the most frequent cause of a HLM is thought to be a post-traumatic injury of the PMFs [9, 20–26].

Clinical and imaging diagnosis of these lesions is always challenging, even though MRI using proton density sequences may be useful [2, 7, 27–29]. In particular, it has been reported that the detection rate of PMFs on a routine knee MRI on sagittal and coronal plane is approximatively 60% [27]. The clinical diagnosis is even more challenging, as most PMFs, tears occur in multi-ligamentosus injuries [14]. When a tear of the PMFs contributes to make the lateral meniscus unstable, arthroscopic observation and probing into the popliteomeniscal fascicle area is helpful to identify the damaged fascicle [16]. In particular, many knees with acute and chronic ACL and/or posterolateral injuries have concurrent damage to the popliteomeniscal fascicles [30]. Tears of PMFs are the most frequent lesion occurring in 80% of patients with grade III posterolateral injuries associated with ACL insufficiency [30–32]. The risk is that an ACL injury could wrongly be identified as the isolated cause of instability and knee pain [6].

Unrecognised tears to the structure comprising the PLC have been cited as an important factor in postsurgical failure after cruciate ligament reconstruction and in chronic instability and degenerative changes after knee trauma [6]. PMFs tears lead to lateral knee pain, painful squatting and locking sensation [9, 11, 16, 18, 20, 24, 30]. Several surgical options are available: from open surgery to arthroscopic ones [2, 3, 32]. This systematic review identifies and analyses the articles on PMFs tears from diagnosis to surgical approach.

## Methods

**Fig. 2:**
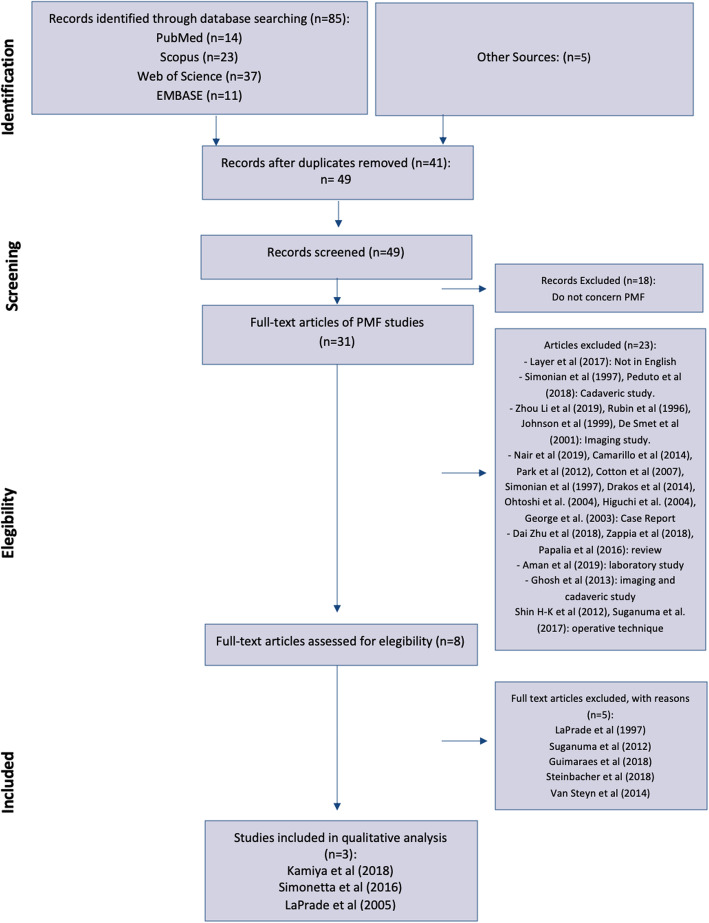
PRISMA flow-chart. Methodology of selection used to screen and include articles for qualitative analysis

The PRISMA (Preferred Reporting Items for Systematic Reviews and Meta-Analyses) guidelines were followed to perform this systematic review [33] (Fig. 2).

### Search strategy

Four electronic databases (PubMed, Scopus, Web of Science and EMBASE) were used to search the scientific literature using various combination of the keywords “Popliteomeniscal Fascicles”, “Lateral Meniscus”, “Popliteal Hiatus”, “Posterolateral Corner”, “Tear” and “Surgical Repair” for the years 1950–2020. The final search was performed on 1 December 2020 by two indipendent investigators. All the resulting titles were organised and screened indipendently. In case of disagreement, a third senior investigator was asked to check and screened the resulting titles.

### Selection criteria

Only studies published in peer-reviewed journals were included. Using the Oxford Center of Evidence-Based Medicine guidelines, level I to IV articles were identified. Studies with patients assessed by pre-operative MRI and then arthroscopically for post-traumatic PMFs tear were included. Furthermore, studies reporting surgical repair of PMFs outcomes assessed by clinical examination and/or subjective evaluation scales were included. Reviews, metanalyses, cadaveric and animal studies, biomechanical studies, case report, commentaries, expert opinions and operative techniques were excluded. We also decided to exclude studies in which no information about the surgical procedure performed, diagnosis, follow-up, pre-operative imaging, arthroscopic or surgical assessment of PMFs and associated tears, pre-operative clinical examination and clinical postoperative outcomes were recorded.

#### Evaluation of the study quality

The methodological quality and bias of each study were evaluated with the Coleman Methodology Score (CMS) [34], which assesses methodology with 10 criteria, giving a total score between 0 and 100. A score of 100 indicates that the study largely avoids chance, various biases, and confounding factors. The subsections that consistute the CMS are based on the subsections of the Consolidated Standards of Reporting Trials (CONSORT) statement (for randomized controlled trials). Each study was scored by two indipendent reviewers and matched each other (Table 1). In case of mismatch, a third investigator was asked to perform the CMS assessment indipendently. Possible disagreements were resolved by discussion.

**Table 1:** Study characteristics

***Study***	***Year of publication***	***Level of evidence***	***Study design***	***Mean follow-up (months)***	***Coleman Methodology Score (CMS)***
Kamiya et al. [20]	2018	IV	Retrospective case series	37	59
Simonetta et al. [18]	2016	IV	Retrospective case series	38.3	46
LaPrade et al. [30]	2005	IV	Retrospective case series	36	43

The most significative data concerning the quality and the study methodology used are reported

### Data extraction

To avoid any bias of selection, the included articles with all the relative list of references, and the articles excluded from the study were reviewed, assessed, and discussed by all the authors. In case of disagreement among the reviewers regarding the selection of articles based on inclusion and exclusion criteria, the senior investigator made the final decision. The following data were independently extracted by all the investigators: demographics, inlcuding mean age, sex, level of activity of population; mean follow-up; timing from symptoms to surgery; pre-operative MRI assessment; associated lesion reported; certainty of diagnosis by pre-operative clinical examination and arthroscopic confirmation; surgical management and technique performed; clinical outcome measurements by post-operative clinical examination and/or subjective evaluation scales; recurrence of the lateral meniscus instability and/or pain and/or locking sensation, and intra- and/or post-operative complications.

## Results

### Study selection

A total of three articles were included in the qualitative analysis. Figure 1 describes the methodology used for selection and inclusion of articles. The original literature search identified a total of 85 articles comprising of duplicates. Another five articles were identified by other sources. After removal of 41 duplicates, 49 articles were assessed for eligibility. Eighteen articles were removed because they did not concern PMF. Of the remaining 31 full-text articles, 22 articles were removed because do not meet inclusion criteria: 1 was excluded because was a review in German language; 2 articles were excluded because they were cadaveric studies; 4 articles were excluded because they were imaging studies; 9 articles were excluded because they were case reports; 3 were excluded because they were reviews of literature; 1 article was a laboratory study; 1 article was a cadaveric and imaging study; 2 articles were excluded because they described operative techniques without patient outcomes. A total of 8 articles were therefore assessed. Finally, three articles were included for the qualitative analysis: LaPrade et al. (2005) [30], Simonetta et al. (2016) [18] and Kamiya et al. (2018) [20]. Five articles were excluded with reasons: LaPrade et al. (1997) [2] was excluded because, although an arthroscopic visualization of PMF tear with other associated lesions of PLC was reported, it is not specified whether and how PMFs were repaired; Steinbacher et al. (2019) [23] and Van Steyn et al. (2014) [22] were excluded because, although they report the surgical management of HLM, in their articles PMFs tears were not visualized and not identified as the cause of HLM; Guimaraes et al. (2018) [8] was excluded because the surgical technique is not discussed; Suganuma et al. (2012) [9] was excluded because, although an arthroscopic confirmation of PMF tears was performed, the surgical technique is not described.

### Study characteristics and quality assessment

All the articles included for the qualitative analysis were published in the period 2005 to 2018, and their characteristics are summarized in the Table 1. All the articles included are retrospective case series, and their level of evidence according to the Oxford Center of Evidence-Based Medicine guidelines is IV. The mean follow-up was 37.1 months, ranging from 36 months [30] to 38.3 months [18]. According to the Coleman Methodology Score (CMS), 2 articles [18, 30] were of poor quality (< 50), and 1 [20] of fair quality (59). The median CMS was 49.3 (43–59) of a possible 100 total score.

### Patient characteristics

**Fig. 3:**
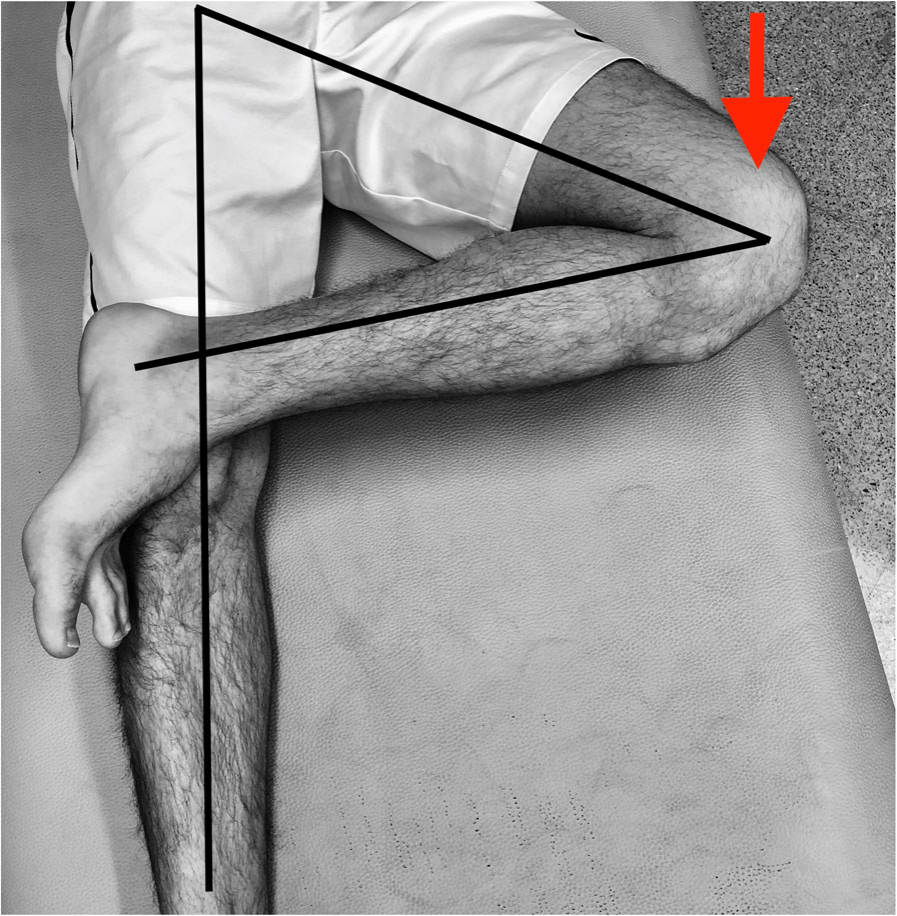
Figure-4 test. Illustration of figure-4 test drawn with black lines indicating the correct position, with the examined knee flexed upon the other leg. The red vector indicates the strength direction to apply to the knee to provoke pain in the lateral compartment

Patient characteristics and surgery indications are reported in Table 2. A total of 32 patients (17 males and 15 females; mean age 26.9 (range 15–67) years) from the included articles was analysed. The level activity was reported for 9 of 32 patients: it ranged from recreational activity to semi-professional and professional sport. In LaPrade et al. case series [30], 2 patients were professional wrestlers, while in Simonetta et al. series [18], 5 were semi-pro soccer players. The diagnosis and the indications for surgery were based on patient symptoms, pre-operative clinical examination and imaging. In all the articles included, patients experienced symptoms such as pain and locking sensation. The pre-operative clinical examination was based on the Figure-4 test [18, 30] (Fig. 3), which was positive in all patients in LaPrade et al. study [30], and in 3 of 6 patients in Simonetta et al. study [18]. Kamiya et al. [20] do not report the results of any clinical examination.

**Table 2:** Patients characteristics and surgical indications

***A. Patient characteristics*** ^***a***^					***B. Surgical indications*** ^***a***^			
	***N of patients***	***Mean age***	***Sex (male/female)***	***Level of activity***	***Symptoms***	***Mean timing symptoms to surgery (months)***	***Pre-operative clinical examination***	***Pre-operative imaging***
Kamiya et al. [20]	20	37.7	9M/11F		Locking sensation	25.3		3D-virtual load MRI
Simonetta et al. [18]	6	16.6	4M/2F	5/6 semi-pro athlete; 1/6 recreational	Pain, locking sensation	14.6	Figure-4 test +: 3/6 patients	MRI in the sagittal plane and T2 sequences
LaPrade et al. [30]	6	26.6	4M/2F	2/6 professional wrestler; 1/6 football player	Pain, locking sensation	5.6	Figure-4 test +: 6/6 patients	MRI

^a^Blank cells indicate not available

On the left side of the table, number of patients, mean age, sex and activity level are summarized; on the right side: symptoms, mean timing from onset of symptoms to surgery, pre-operative clinical examination and imaging are shown

The imaging study to diagnose PMF tears was MRI in for all included articles. Kamiya et al. [20] performed a 3D-virtual load MRI, which allows, under dynamic load, to verify the forward and medial translation of the lateral meniscus during knee flexion. Simonetta et al. [18] imaging study was based on MRI, and the diagnosis of PMF tear was confirmed in the sagittal plane and T2 sequences that demonstrate the disruption of PMFs. LaPrade et al. [30] used plain MRI scan to demonstrate PMF tear. The mean timing of symptoms to surgery, reported in all the articles, was 15.1 months (range 0.3–60).

### Surgical approach and suturing technique

In Table 3, the surgical technique is reported. To confirm the diagnosis and assess the disruption of the PMF, arthroscopic evaluation is necessary. In all the articles included, direct arthroscopic visualization confirmed the PMF tear between the posterior horn of the lateral meniscus and the popliteus hiatus.

**Table 3:** Surgical characteristics

	***Arthroscopic confirmation***	***Surgical procedure***	***Suturing technique***	***Associated lesion***
Kamiya et al. [20]	Direct visualization of disruption of PMF. Forward translation by probing intact lateral meniscus	Inside-out arthroscopic repair	Polyester non-absorbable sutures on a 10-inch straight and/or curved cutting needle (Stryker). An average of 5.0 (range, 2–8) double-stacked vertical suture were used.	Isolated PMFs
Simonetta et al. [18]	Direct visualization of disruption of PMF. Forward translation by probing intact lateral meniscus	All-inside arthroscopic repair	Fastfix meniscal repair system (Smith & Nephew) Two to three sutures are placed on either side of the popliteal hiatus, in a vertical fashion	4/6 chondral lesions LFC
LaPrade et al. [30]	Direct visualization of disruption of PMF. Forward translation by probing intact lateral meniscus	Open repair	horizontal mattress non-absorbable 0 sutures	Isolated PMFs

Details of arthroscopic confirmation of the diagnosis, surgical and suturing technique used, and the associated lesion reported during the procedures

Further evidence of PMF tear was given by the increased mobility and forward translation of the intact lateral meniscus on arthroscopic probing.

Different surgical procedures are reported: both Kamiya et al. [20] and Simonetta et al. [18] performed an arthroscopic technique, while LaPrade et al. [30] performed an open repair. Each article reports a different suturing technique.

Kamiya et al. [20] performed an inside-out repair of injured PMFs using a polyester non-absorbable suture on a 10-inch straight and/or curved cutting needle (Stryker, Kalamazoo, CA, USA). An average of 5.0 (range 2–8) double-stacked vertical stitches connecting the lateral meniscus and the meniscocapsular junction was reported. No other associated lesions were noted at the arthroscopic evaluation.

Simonetta et al. [18] used the FastFix meniscal repair system (Smith & Nephew, Hull, UK) to fix disrupted PMFs with an all-inside technique. Two or three stitches in a vertical fashion on either side of the popliteal hiatus were used. In their case series, 4 of 6 patients, in whom timing from symptoms to surgery was greater than 12 months, presented a chondral lesion of the lateral femoral condyle, but these associated lesions were not addressed.

La Prade et al. [30] performed an open repair. For this purpose, after an antero-lateral capsular arthrotomy, the midthird lateral capsular ligament was retracted and the antero-inferior fascicle tear was exposed. After this, an open repair using horizontal mattress sutures with non-absorbable 0 sutures was performed.

### Outcome analysis

Clinical outcome with evaluation subjective scales, peri-operative complications and post-operative imaging studies are reported in Table 4. For all three articles included, the clinical evidence at the final follow-up demonstrated a complete resolution of pre-operative symptoms (pain and locking feeling), but only LaPrade et al. [30] recorded at the final follow-up the result of clinical post-operative examination, demonstrating a negative figure-4 test for all their patients. No subjective scales were used for outcome assessment except for Kamiya et al. [20], who recorded a mean Tegner activity level before and after surgery of 4.6 (range, 2–8) and 4.7 (range, 2–8), respectively (*p* = 0.480). The Lysholm knee score significantly increased from 72.0 (range, 48–85) to 97.8 (range, 78–100) (*p* < 0.01)*.* None of the analysed studies reported clinically relevant complications. Post-operative imaging assessment was performed only by Kamiya et al. [20] using a 3D-virtual load MRI at 4-month post-operatively, demonstrating the absence of a mobile lateral meniscus during knee flexion with a successfull repair of PMFs.

**Table 4:** Outcome^a^. Symptoms and post-operative clinical evaluation, evaluation scales, complications and post-operative imaging study

	***Clinical outcome***	***Evaluation scales***	***Complications***	***Post-operative imaging***
Kamiya et al. [20]	Absence of locking and pain at final F-U	The mean Tegner activity level scales before and after surgery was 4.6 (range, 2–8) and 4.7 (range, 2–8), respectively (*p* = 0.480). The Lysholm knee scores were significantly increased from 72.0 (range, 48–85) to 97.8 (range, 78–100) (*p* < 0.01)	None	3D-Virtual load MRI after 4 months
Simonetta et al. [18]	Absence of locking and pain at final F-U		None	
LaPrade et al. [30]	Figure-4 test -:6/6 Absence of locking and pain at final F-U		None	

^a^Blank cells indicate not available

## Discussion

The diagnosis, by imaging and clinical examination or arthroscopically, of disrupted PMFs remains very challenging [18, 32]. Tears of PMFs are present in 80% of patients with an ACL tear or traumatic injury to the PLC [8, 30, 32, 35]. In symptomatic patients, surgery is the gold standard to manage these injuries [21, 36], although the most appropriate surgical technique is still debated [3, 22, 30, 37]. This systematic review shows surgery for symptomatic patients with PMF tears is effective and safe, with pain relief and resolution of locking sensation with no complications reported. Furthermore, although the diagnosis is not easy and an accurate clinical examination and imaging, by MRI scan, is fundamental to plan the most appropriate treatment, arthroscopic evaluation by probing the lateral meniscus and the intra-articular visualization of disrupted PMFs remain the gold standard to accurately identify these injuries. The lack of high level-of-evidence studies about this topic and the paucity of articles regarding the management of PMFs tears does not allow to fully clarify which could be the most appropriate surgical technique to undertake.

According to our results, younger patients with high-demanding activities are most suitable candidates for surgery. Although Kamiya et al. [20] operated on patients older than 50, the mean age among the 32 patients analysed was 26.9. Only for 9 patients the level of activity was specified [18, 30]: 7 of 9 patients were professional or semi-professional athletes. High-demanding athletic patients are susceptible to PMFs tears, with the common injury mechanism being twisting on their knee. Full-contact sports, such as wrestling or soccer, are the most commonly involved.

The indications for surgery included symptoms, clinical examination, except for Kamiya et al. [20], and imaging: pain over the lateral aspect of the knee, pain during active joint flexion and a locking sensation were reported in all the patients described in the studies included in the present review. A specific clinical examination was performed by LaPrade et al. [30] and Simonetta et al. [18]: the figure-4 test, first described by LaPrade [2], was chosen as diagnostic test to check the integrity of PMF. Of 12 patients undergoing the figure-4 test, 9 (75%) resulted positive, indicating that a positive figure-4 test is strongly suggestive of PMF tear. In all the patients reported, the diagnosis of PMF disruption was confirmed arthroscopically. Arthroscopy is crucial to check the lateral meniscus stability [11, 18, 20, 22–24, 26, 30, 36]. If the PMFs are disrupted, probing the lateral meniscus produces an abnormally large forward translation of the meniscus with a direct visualization of PMF tear on the lateral side of the joint space. In LaPrade et al. [30] study, although they performed an open repair, an arthroscopic check to confirmation the diagnosis was performed. The time from onset of symptoms to surgery suggests that timing does not influence the healing potential of PMF, and that the continuous forward movement of the lateral meniscus prevents the formation of a scar tissue that would stabilise the meniscus. PMF tears are often associated with other intra-articular injuries such as ACL tears, meniscal and chondral tears, and PLC injuries [2, 3, 8, 32]. Furthermore, associated chondral lesions of the lateral femoral condyle were reported in 4 of 32 (12.5%) patients. These four patients had reported symptoms for longer than 12 months. To prevent further damage to the knee joint and protect other structures which have been injured or reconstructed (ACL, meniscal and chondral lesions) or stabilize the lateral compartment, prompt surgery in active patients with symptoms and a positive MRI and clinic examination is mandatory.

A pre-operative MRI is always performed and appears the best imaging modality to demonstrate disruption of PMF on the sagittal plane and in T2 sequences [7, 8, 29]. Kamiya et al. [20] performed a virtual 3D-loading MRI which demonstrated the forward translation of the lateral meniscus that during active knee flexion, a finding subsequently confirmed arthroscopically.

The appropriate surgical technique is still debated [3, 18, 30, 32]. In all the articles included in the present review, the surgical procedure is well described and characterized. While Kamiya et al. [20] and Simonetta et al. [18] performed two different arthroscopic techniques, an inside-out and an all-inside repair respectively, LaPrade et al. [30] choose an open repair, with three different suturing technique. All three articles reported good clinical outcome with resolution of symptoms and no complications: only LaPrade et al. [30] performed a new clinical examination by subjecting patients to a post-operative figure-4 test, which always resulted negative. Kamiya et al. [20] reported subjective evaluation scales and post-operative MRI imaging study: the Tegner and Lysholm evaluations scores showed an improvement between pre- and post-operative values.

### Study limitations

The limitations of the present systematic review are related to the scanty quality of the studies available in the literature. All the articles included were retrospective case series, with limited number of patients. Furthermore, the absence of subjective evaluation scales and no post-operative assessment by MRI imaging, except for Kamiya et al. [20], do not allow complete outcome analysis. The level of evidence for all the articles included was low (IV). According to the Coleman Score, the study quality ranges from fair [20] to poor [18, 30].

## Conclusion

Although the number of studies available in the literature was limited and the methodological quality of the articles included was questionable, the most relevant evidence arising from this systematic review is that it is important to recognise PMF disruption in patients with pain and locking sensation, and no clear evidence of other causes of internal derangement of the knee. In particular, it is necessary to evaluate PMF tears by pre-oeprative MRI imaging, and clinical examination using the figure-4 test. Arthroscopic confirmation of the diagnosis by direct visualization of the PMF tear and probing of the lateral meniscus is necessary. When recognised, the evidence suggests to operate on the lesion to stabilise the knee joint, especially in young athletic patients. There is no definite evidence about the most appropriate surgical technique to perform, and surgeon experience and confidence should guide the choice of the appropriate technique. Further studies with a higher number of patients, and higher methodological quality should be undertaken to better understand the pathogenesis and the evolution of PMF tears and to clarify the best surgical technique.

## Data Availability

The datasets used and/or analysed during the current study are available from the corresponding author on reasonable request.

## Abbreviations

PMFs: Popliteomeniscal fascicles
PLC: Postero-lateral corner
HLM: Hypermobile lateral meniscus
MRI: Magnetic resonance imaging
ACL: Anterior cruciate ligament
CMS: Coleman Methodology Score
